# Low Expression of Programmed Death 1 (PD-1), PD-1 Ligand 1 (PD-L1), and Low CD8+ T Lymphocyte Infiltration Identify a Subgroup of Patients With Gastric and Esophageal Adenocarcinoma With Severe Prognosis

**DOI:** 10.3389/fmed.2020.00144

**Published:** 2020-04-28

**Authors:** Silvio Däster, Serenella Eppenberger-Castori, Valentina Mele, Hannah M. Schäfer, Lukas Schmid, Benjamin Weixler, Savas D. Soysal, Raoul A. Droeser, Giulio C. Spagnoli, Christoph Kettelhack, Daniel Oertli, Luigi Terracciano, Luigi Tornillo, Urs von Holzen

**Affiliations:** ^1^Department of Surgery, University Hospital Basel, Basel, Switzerland; ^2^Division of Molecular Pathology, Institute of Pathology, University Hospital Basel, Basel, Switzerland; ^3^Department of Biomedicine, University of Basel, Basel, Switzerland; ^4^National Research Council, Institute of Translational Pharmacology, Rome, Italy; ^5^Harper Cancer Research Institute, Indiana University School of Medicine South Bend, South Bend, IN, United States; ^6^Goshen Center for Cancer Care, Goshen, IN, United States

**Keywords:** gastric cancer, esophageal cancer, PD-1, PD-L1, CD8+ lymphocyte infiltration

## Abstract

Prognosis of gastric and esophageal cancer is poor and treatment improvements are needed. Programmed cell death 1 receptor (PD-1) interaction with its ligand PD-L1 in tumor micro-environment promotes immune tolerance and blocking monoclonal antibodies have entered clinical practice. However, clinical significance of PD-1 and PD-L1 expression in gastric and esophageal adenocarcinomas, particularly in non-Asian patients, is still unclear. Three tissue microarrays including 190 clinically annotated esophageal (*n* = 31) and gastric (*n* = 159) adenocarcinomas and 58 paired mucosa specimens, were stained with PD-1, PD-L1, and CD8-specific reagents in indirect immunohistochemistry assays. PD-L1 expression was detectable in 23.2% of cancer specimens. High PD-1 expression was detectable in 37.3% of cases and high CD8+ infiltration in 76%. PD-L1 and high PD1 expression significantly correlated with each other (*r*_s_ = 0.404, *P* < 0.0001) and both significantly correlated with CD8+ infiltration (*r*_s_ = 0.435, *P* = 0.0003, and *r*_s_ = 0.444; *P* = 0.0004, respectively). CD8+ lymphocyte infiltration correlated with improved survival in univariate (*P* = 0.009), but not multivariate analysis. Most interestingly, multivariate analysis and Kaplan-Meier curves indicate that combined low PD-1/PD-L1 expression and low CD8+ lymphocyte infiltration significantly correlate with poor prognosis. Our data document the clinical significance of a microenvironmental signature including PD-1/PD-L1 expression and CD8+ lymphocyte infiltration in gastric and esophageal adenocarcinomas and contribute to identify a patients' subset requiring more aggressive peri-operative treatments.

## Introduction

Gastric and esophageal cancers are major causes of cancer-related death. While esophageal cancer is the sixth most common cause of cancer-related death worldwide, gastric cancer is the second, with a high incidence in Asian countries (1). Localized tumors are routinely treated with surgical resection. However, even after potentially curative resection, overall survival (OS) is poor (2, 3), and innovative therapeutic approaches are needed.

Interaction of programmed death 1 (PD-1) immune checkpoint protein with its ligands PD-L1 and PD-L2 inhibits anticancer immune responses (4, 5). Therefore, monoclonal antibodies (mAb) preventing it are currently utilized in cancer treatment (6–11). The use of immune checkpoint inhibitors has also been suggested for gastric and esophageal cancer treatment and different clinical trials have shown promising results (12–16), as recently reviewed (17). Notably, the anti-PD-1 antibody nivolumab (Opdivo®) is approved in Japan as third-line treatment for gastric cancer and the anti-PD-1 antibody pembrolizumab (Keytruda®) recently received FDA approval for the treatment of patients with PD-L1 positive gastric and gastroesophageal junction adenocarcinoma.

Responsiveness to immunotherapy critically depends on the characteristics of tumor immune contexture (18, 19). Therefore, it is important to investigate CD8+, PD-1+, and PD-L1+ cell infiltration in large numbers of clinical specimens. Yet, the few published studies addressing gastric and esophageal cancer frequently yield conflicting results. Importantly, due to the high incidence of these cancers, most studies are from East Asia. However, gastric and esophageal carcinomas in Asian and non-Asian patients are characterized by different clinical course and immune infiltration (20). Moreover, molecular subtypes may be characterized by different microenvironment composition and expression of checkpoint molecules (21, 22). Therefore, studies from different geographic areas are necessary.

Due to limited number of reports from Western countries and their low patient numbers, there is still a paucity of data regarding prognostic relevance, and therapeutic potential of CD8+ and PD-1+/PD-L1+ infiltration in gastric and esophageal cancers. In this study, we addressed these issues by using tissue microarray (TMA) technology.

Whole-genome sequence studies classify gastric adenocarcinoma (GAC) into four different subtypes: (i) Epstein-Barr virus (EBV) positive tumors, characterized by PD-L1/-L2 amplification, (ii) microsatellite unstable (MSI) cancers, (iii) tumors with chromosomal instability, and (iiii) genomically stable tumors (22). In esophageal cancers, three molecular subclasses of squamous cell carcinomas (ESCC) were identified, whereas adenocarcinomas (EAC) closely resemble chromosomal unstable gastric cancers (21). Most recently, EBV+ and MSI gastric cancers have been shown to respond to anti PD-1 treatment (23).

Since our earlier studies (Däster et al., unpublished data) indicate that ESCC are poorly infiltrated by immune cells, and considering their highly specific molecular features, in this study we focused on the analysis of gastric and esophageal adenocarcinomas.

## Materials and Methods

### Tissue Microarray Construction

Three TMA blocks of non-consecutive primary esophageal and gastric cancer and paired non-malignant adjacent tissue specimens were constructed by using TMA-Grand Master® (3DHisteck, Sysmex AG, Switzerland). TMAs contained 1 mm cores of 190 gastric and esophageal adenocarcinomas together with 58 non-malignant mucosa samples. All specimens were part of the Biobank at the Institute of Pathology, University Hospital Basel, Switzerland. For TMA construction, formalin-fixed, paraffin-embedded tissue blocks were prepared according to standard protocols. All cancer cores were checked for their percentage of tumor cells and this value was above 50% for all cancer samples.

### Data Collection

Clinical and histopathology data were collected retrospectively in a non-stratified and non-matched manner. Data included patients' age, primary cancer site, TNM stage, disease-specific survival, histological subtype, presence of vascular invasion, and tumor diameter and grade. Approval for the use of samples and data was obtained from local ethics committee (Permission 361/12 EKBB).

### Immunohistochemistry

All analyses have been performed on Ventana BenchMark Ultra. Primary antibodies used were specific for CD8 (Ventana 790-4460 clone sp57, Roche, Switzerland), PD-1 (Ventana 760-4895, Roche, Switzerland), and PD-L1 (Ventana SP263 assay, Roche, Switzerland). According to manufacturer's OptiView procedures, the following pre-incubation and first mAb times were utilized: 24 and 16min, 48 and 12 min, 56 and 12min for anti-CD8, -PD1, and -PD-L1, respectively. Immunohistochemistry (IHC) evaluation was performed by a senior consultant pathologist [LTO]. Intraepithelial and stromal CD8+ cells were counted for each TMA punch (approximately one high power [20x] field). For PD-1 and PD-L1 staining, percentages of positive cells/total number of cells, and staining intensities (0 = negative, 1 = weak, 2 = moderate, 3 = strong) were considered for each TMA punch. PD-1-positivity was only detectable in tumor infiltrating lymphocytes, whereas PD-L1-positive cells included tumor and immune cells, which were evaluated in combination. Subsequently, PD-1 and PD-L1 histoscores were obtained by multiplying percentages of positive cells by staining intensity.

### Statistical Analysis

Data were analyzed using the Statistical Package Software R (Version 3.4.1, www.r-project.org). Descriptive statistics included mean ± standard deviation for parameters with Gaussian distribution or percentage of frequencies for occurrences for discrete variables. Cut-off values used to classify tumors with low or high infiltration of positive cells/histoscores were calculated by regression tree analysis (rpart package). Thereafter, based on the results (high/low) of all three markers, tumors were classified into three groups “PD-1/PD-L1/CD8 high” (high PD-1 and PD-L1 histoscores, high infiltration of CD8+ cells), “PD-1/PD-L1/CD8 low” (low PD-1 and PD-L1 histoscores, low infiltration of CD8+ cells), and “PD-1/PD-L1/CD8 mixed” (the remaining tumors).

Chi-square, Fisher's exact, Mann-Whitney-Wilcoxon, Kruskal-Wallis, and Jonckheere-Terpstra tests were used to determine the association of CD8, PD-1, and PD-L1 positivity and clinical-pathological features. Relationships between continuous markers were calculated using Spearman's correlation.

Survival analysis was carried out by Cox regression analysis and Kaplan-Meier curves were compared by log rank test. Any missing clinical-pathological information was assumed to be missing at random. Multivariate Cox regression analysis and hazard ratios (HR) and 95% confidence intervals (CI) were used to determine prognostic effects on survival. *P* ≤ 0.05 were considered statistically significant.

## Results

### Patient and Tumor Characteristics

Clinical pathological characteristics of patients under investigation (*n* = 190) are reported in Table 1. Tissue samples from 31 esophageal adenocarcinoma to 10 paired non-malignant esophageal biopsies, as well as 159 gastric cancers and 48 non-malignant paired gastric tissue biopsies were evaluated. Four EAC were gastroesophageal junction tumors. Over 75% of tumors were in T2-3 stage and over 70% were in N0-1 stage. A majority of tumors were characterized by a G3 histological grade.

**Table 1:** Clinical-pathological characteristics of the overall gastric and esophageal adenocarcinoma patient cohort (*n* = 190).

Characteristics	
Patients' age mean/median (range)	69/71 (27–90)
Tumor size in mm mean/median (range)	54/45 (10–180)
Localization	
Esophagus	27 (14.2%)
Esophago-gastric junction	4 (2.1%)
Stomach	159 (83.7%)
Sex	
Female	58 (30.5%)
Male	132 (69.5%)
T stage	
T1	26 (13.7%)
T2	68 (37.9%)
T3	72 (37.9%)
T4	24 (12.6%)
N stage*	
N0	61 (32.1%)
N1	78 (41.1%)
N2	27 (14.2%)
N3	22 (11.6%)
Tumor grade**	
G1	7 (3.7%)
G2	52 (26.4%)
G3	115 (63.4%)
Vascular invasion	
No (%)	26 (13.7%)
Yes (%)	78 (41.5%)
unknown	86 (44.8%)

* *Data not available for 2 patients*.

** *Data not available for 16 patients*.

Median overall survival (OS) time in the whole cohort was 8 months (range 0–191) and 5-year OS rate was 51.0% (95% CI = 41.6–62.5).

### CD8, PD-1, and PD-L1 Expression in Gastric and Esophageal Adenocarcinoma Specimens

**Figure 1:**
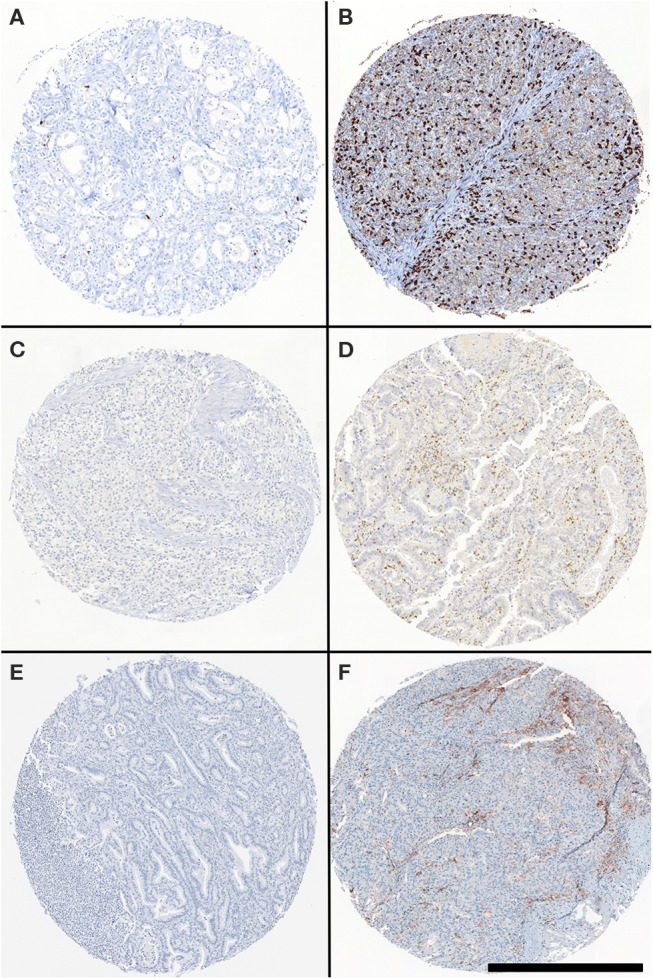
CD8-, PD-1-, PD-L1 specific staining in gastric and esophageal adenocarcinoma TMA. Representative CD8, PD-1, and PD-L1-specific staining in TMA punches. Specimens were stained with CD8- **(B)**, PD-1- **(D)**, and PD-L1- **(F)** specific reagents. **(A, C, E)** refer to punches stained with isotype control reagents. Scale bar: 500 μm.

Representative examples of CD8, PD-1, and PD-L1-specific staining are shown in Figure 1. Total number of CD8+ lymphocytes detectable within tumor and stroma ranged between 0 and 548 (mean = 95 ± 9.6, median = 66). Tissue specimens were dichotomized as highly (76.2%) or poorly (23.8%) infiltrated, based on a threshold value of 33 CD8+ cells per 1 mm diameter punch, as identified by regression tree analysis. This value also corresponds to the first quartile of infiltrating CD8+ lymphocytes counted in this cohort. The calculated histoscore for PD-1+ (percentage of positive cells multiplied by intensity) in gastric and esophageal adenocarcinoma samples ranged between 0 and 210 (mean = 23 ± 39, median = 4) and a threshold value of 14 was calculated. PD-L1 histoscore, considering all positive cells in each punch, ranged between 0 and 160 (mean = 6.8 ± 21, median and third quartile = 0). Therefore, samples were scored as positive when at least one cell was specifically stained. Thus, a positive signal was detectable in 43/185 tumors (23.2%), but in no normal tissue.

Notably, a moderately positive correlation was observed between PD-1+ and PD-L1 histoscores (r_s_ = 0.404; *P* < 0.0001) and between each of these scores and CD8+ cell infiltration (*r*_s_ = 0.444; *P* < 0.0004 and *r*_s_ = 0.435; *P* = 0.0003, respectively).

While there was no significant difference in CD8+ infiltration between cancers and normal mucosa samples (*P* = 0.480), a significantly higher number of cancer samples was characterized by higher PD-1 histoscore, as compared to paired normal tissues (*P* < 0.0001) (Supplementary Figure 1).

### Univariate and Multivariate Analysis of CD8, PD-1, and PD-L1 Expression in Gastric and Esophageal Adenocarcinomas

We initially analyzed the prognostic significance of the expression of individual markers. Univariate Cox regression analysis indicated that CD8+ infiltration was highly significantly (*p* = 0.009) associated with improved 5 years OS (Table 2). Instead, higher PD-1 and PD-L1 scores were barely significantly associated “per se” with OS (*P* = 0.056 and *P* = 0.05, respectively). Notably however, a tumor microenvironment “signature” including high CD8+ cell infiltration and high PD1/PD-L1 scores was associated with significantly higher OS (*P* = 0.005). As expectable, pN stage (pos. vs. neg.) appeared to significantly impact on OS (*P* = 0.01).

**Table 2:** Uni- and multivariate Hazard Cox regression survival analysis in the whole cohort of gastric and esophageal cancers.

	**Univariate**			**Multivariate**		
	**HR**	**95% CI**	***p*-values**	**HR**	**95% CI**	***p*-values**
CD8*	0.41	0.21–0.80	0.009			
PD-1 histoscore*	0.47	0.21–1.02	0.056			
PD-L1 histoscore*	0.39	0.15–1.00	0.050			
PD1 PDL1 CD8 Score**	0.44	0.25–0.78	0.005	0.53	0.29–0.96	0.037
Age	0.99	0.97–1.02	0.798	1.01	0.98–1.04	0.623
Gender (men vs. women)	1.36	0.67–2.75	0.392	1.27	0.51–3.21	0.601
pT stage (T3-4 vs. T1-2)	1.72	0.94–3.15	0.076	1.66	0.82 – 3.40	0.160
Tumor grade (high vs. low)	1.17	0.63–2.16	0.623	0.84	0.39–1.82	0.647
pN stage (pos. vs. neg.)	2.49	1.24–5.01	0.010	3.82	1.51–11.09	0.008

*Uni- and multivariate Cox-regression analyses showing Hazard Ratios and P-values (Wald test)*.

* *Not included in the multivariate model*.

** *All three markers low, mixed or all high*.

Upon multivariate analysis, as expectable, pN stage (pos. vs. neg.) emerged as critical variable significantly associated with poor OS (HR:3.82; 95%CI:1.51–11.09; *P* = 0.008) (Table 2). Most interestingly however, low CD8+ lymphocyte infiltration coupled with low PD-1/PD-L1 scores (PD-1/PD-L1/CD8 low) also significantly correlated with poor OS in gastric and esophageal adenocarcinomas (HR;0.53; 95%CI:0.29–0.96; *P* = 0.037). Separate analysis of gastric and esophageal adenocarcinomas is reported in Supplementary Tables 1A,B.

### Impact of PD-1/PD-L1/CD8 Signature in Gastric and Esophageal Adenocarcinomas

Subsequently, we explored clinical-pathological features in the three subgroups identified by uniformly high or low CD8+ infiltration and PD1/PD-L1 scores (PD-1/PD-L1/CD8 high and PD-1/PD-L1/CD8 low) or mixed results (PD-1/PD-L1/CD8 high and/or low). Complete follow-up data were available for 161 patients, including 133 gastric, and 28 esophageal adenocarcinomas. PD-1/PD-L1/CD8 high signature was detectable in slightly older patients as compared with the mixed or low signature (*p* = 0.046), but was independent from patients' gender, tumor size, tumor grade, and pN (data not shown).

**Figure 2:**
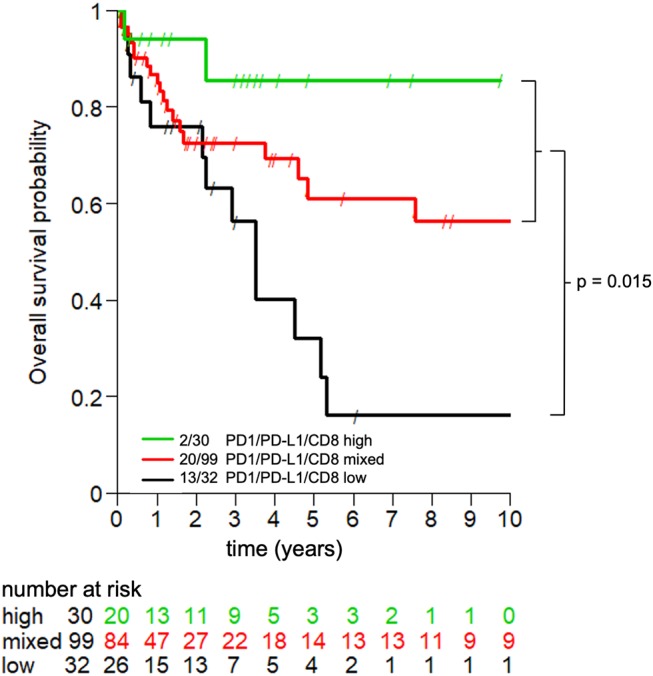
Prognostic significance of PD-1/PD-L1/CD8 signature in gastric and esophageal adenocarcinoma. Kaplan-Meier curves depict the impact of the consistently high (green line), low (black line), or mixed (red line) expression of the indicated markers in tumor microenvironment on the overall survival of patients with gastric and esophageal adenocarcinomas. Long-term follow-up data were available for 161 patients. *P* = 0.015.

Most importantly, survival analysis indicates that gastric and esophageal adenocarcinomas with PD-1/PD-L1/CD8 “low” signature is characterized by poor long-term prognosis, as compared with the other two subgroups under investigation (*p* = 0.015, Figure 2). In particular, PD-1/PD-L1/CD8 “low” signature is characterized by poor long-term prognosis, as compared to the “high” (*p* = 0.008) or mixed signature group (*p* = 0.03), in spite of an apparent initial overlap of the survival curve of the latter. In contrast, due to relatively low numbers of patients under investigation, difference in survival curves of patients with “high” or “mixed” signature failed to reach statistical significance threshold (*p* = 0.2). A separate analysis of Kaplan-Meier curves for gastric and esophageal adenocarcinomas is reported in Supplementary Figures 2A,B.

## Discussion

PD-1/PD-L1 axis plays a crucial role in inhibiting T lymphocyte functions, allowing cancer cells to escape adaptive immune response (4, 5). It has been suggested that “inflamed” tumors with high CD8+ T cell infiltration and high PD-1/PD-L1 expression might most benefit from PD-1/PD-L1 blockade (18).

Due to higher epidemiological relevance, gastric, and esophageal cancers have mainly been studied in Asian cohorts, and there are only few reports focusing on tumor microenvironment in non-Asian patients. However, particularly regarding gastric cancers, distinct gene expression profiles were detected in tumors from Asian and Western cohorts with significantly higher expression of T cell markers in the Western population (20).

In studies focusing on East Asian patients, PD-L1 was shown to be expressed in up to 40% of tumor cells in esophageal cancers, but its prognostic significance is debated (24–27). In a cohort of Western patients, PD-L1 expression was reported to be associated with a favorable clinical course (28).

In gastric cancers, most studies from Asian patients demonstrated immunohistochemical PD-L1 expression in around 40% of tumors and an association with poor survival (29–33), as supported by a recent meta-analysis (34). However, association with favorable prognosis has also been reported (35).

In a Western population of 465 gastric cancer patients, PD-L1 expression was detected in 30% of tumor cells and was associated with better outcome (36). However, association with adverse prognosis was also reported by others (37).

Prognostic significance of PD-1 expression in esophageal and gastric cancers is also debated (27, 29, 30, 35–37).

Regarding CD8+ T cells, current understanding is that high infiltration correlates with improved outcome, as shown in both esophageal (38, 39) and gastric cancers (20, 40–42) in both Asian and non-Asian patients. However, conflicting data have also been published and a recent report in gastric cancer has surprisingly suggested an association between high CD8+ T cell density and poor survival (37). Importantly, CD8 expression was also associated with PD-L1 expression in that cohort (37).

Interestingly, contrasting data have been reported regarding the extent of CD8+ lymphocyte infiltration in these cancers, as compared to corresponding healthy tissues, possibly reflecting differences related to different tumor subtypes (38, 43–46) and Helicobacter pylori status. In our study, we did not observe significant differences in cancer, as compared to healthy tissue CD8+ T cell infiltration. However, consistent with previous reports (44–46), in our series PD-1 expression was increased in cancerous, as compared to normal tissues, whereas PD-L1 expression was undetectable in healthy tissues.

Our data document that although CD8+ T cell infiltration, and, to lower extents, PD-1/PD-L1 expression appear to be associated with improved 5 years OS in univariate analysis, none of these markers significantly correlates “*per se*” with survival in multivariate analysis. Most interestingly however, a combined immunohistochemical analysis reveals that low PD-1/PD-L1/CD8 expression in gastric and esophageal adenocarcinoma microenvironment is associated with poor prognosis. Therefore, patients bearing these tumors should be eligible for early and more aggressive peri-operative treatments.

Our study has limitations. First, TMA technology does not mirror tumor tissue heterogeneity. However, TMA punches included at least 50% tumor cells and were taken from the center of tumor specimens. Moreover, the numbers of individual gastric and esophageal adenocarcinoma specimens compensate, at least in part, for the heterogeneity of different tumor areas. Secondly, the number of patients suffering from esophageal adenocarcinoma is limited. Subsequently the message of this study is consistent mainly for gastric cancers. Lastly, the retrospective design of the study limits its significance. Nevertheless, these data might help developing further prospective and mechanistic studies and contribute to the identification of subgroups of high-risk patients.

## Data Availability Statement

The datasets generated for this study are available on request to the corresponding author.

## Ethics Statement

Approval for the use of samples and data was obtained from local ethics committee (Ethikkommission beider Basel EKBB, Permission 361/12 EKBB).

## Author Contributions

SD, SE-C, GS, and UH contributed conception and design of the work. SD, VM, HS, LS, BW, and CK collected the data. LTe and LTo analyzed the stainings. SE-C organized the database and performed the statistical analysis. SD, SE-C, SS, RD, GS, CK, DO, and UH were responsible for data analysis and interpretation. SD wrote the first draft of the manuscript. SE-C and GS wrote sections of the manuscript. All authors contributed to manuscript revision, read, and approved the version to be published.

## Conflict of Interest

The authors declare that the research was conducted in the absence of any commercial or financial relationships that could be construed as a potential conflict of interest.
