# Supplementary material for: Low Expression of Programmed Death 1 (PD-1), PD-1 Ligand 1 (PD-L1), and Low CD8+ T Lymphocyte Infiltration Identify a Subgroup of Patients With Gastric and Esophageal Adenocarcinoma With Severe Prognosis
Source: Front Med (Lausanne). 2020 Apr 28;7:144. doi: 10.3389/fmed.2020.00144 (PMC7199486; doi:10.3389/fmed.2020.00144)
Supplement: Supplementary file 1 [file Data_Sheet_1.docx]

**Supplementary materials**

Low expression of programmed death 1 (PD-1), PD-1 ligand 1 (PD-L1), and low CD8+ T lymphocyte infiltration identify a subgroup of patients with gastric and esophageal adenocarcinoma with severe prognosis

Däster S, Eppenberger-Castori S, Mele V et al.

**Supplementary Table 1A:** Uni- and multivariate Hazard Cox regression survival analysis in patients with gastric adenocarcinoma. Data from 159 tumors were available.

|  | **Univariate** | | | **Multivariate** | | |
| --- | --- | --- | --- | --- | --- | --- |
|  | **HR** | **95% CI** | **p-values** | **HR** | **95% CI** | **p-values** |
| **CD8*** | 0.34 | 0.15 – 0.79 | **0.011** |  |  |  |
| **PD-1 histoscore*** | 0.35 | 0.12 – 1.01 | **0.052** |  |  |  |
| **PD-L1 histoscore*** | 0.52 | 0.20 – 1.26 | 0.148 |  |  |  |
| **PD1 PDL1 CD8 Score**** | 0.26 | 0.09 – 0.70 | **0.004** | 0.44 | 0.20 – 0.95 | **0.037** |
| **Age** | 0.99 | 0.96 – 1.02 | 0.578 | 0.99 | 0.96 – 1.02 | 0.613 |
| **Gender (men vs women)** | 1.22 | 0.58 – 2.56 | 0.603 | 1.11 | 0.43 – 2.92 | 0.827 |
| **pT stage (T3-4 vs T1-2)** | 2.16 | 1.05 – 4,4 | **0.036** | 1.98 | 0.85 – 4.66 | 0.115 |
| **Tumor grade (high vs low)** | 1.16 | 0.57 – 2.37 | 0.680 | 0.90 | 0.36 – 2.26 | 0.829 |
| **pN stage (pos. vs neg.)** | 2.37 | 1.09 – 5.12 | **0.028** | 3.37 | 1.13 – 10.04 | **0.029** |

Uni- and multivariate Cox-regression analyses showing Hazard Ratios and *P*-values (Wald test).

*Not included in the multivariate model

** All three markers low, mixed or all high

**Supplementary Table 1B:** Univariate Hazard Cox regression survival analysis in patients with esophageal adenocarcinomas. Data from 28 tumors were available. Multivariate analysis could not be performed due to the low case number.

|  | **Univariate** | | |  | | | |
| --- | --- | --- | --- | --- | --- | --- | --- |
|  | **HR** | **95% CI** | **p-values** |  | |  |  |
| **CD8** | 0.80 | 0.24 – 2.64 | 0.719 |  |  | |  |
| **PD-1 histoscore** | 0.59 | 0.17 – 2.05 | 0.415 |  |  | |  |
| **PD-L1 histoscore*** |  |  |  |  |  | |  |
| **PD1 PDL1 CD8 Score**** | 0.25 | 0.03 – 1.98 | 0.190 |  |  | |  |
| **Age** | 1.02 | 0.96 – 1.08 | 0.578 |  |  | |  |
| **Gender (men vs women)*** |  |  |  |  |  | |  |
| **pT stage (T3-4 vs T1-2)** | 0.91 | 0.28 – 2.86 | 0.865 |  |  | |  |
| **Tumor grade (high vs low)** | 1.23 | 0.36 – 4.23 | 0.739 |  |  | |  |
| **pN stage (pos. vs neg.)** | 3.51 | 0.44 – 27.9 | 0.235 |  |  | |  |

Univariate Cox-regression analysis showing Hazard Ratios and *P*-values (Wald test).

*Missing data

** All three markers low, mixed or all high

**Supplementary figure 1.**

CD8+ lymphocyte infiltration and PD-1 histoscore in gastric and esophageal adenocarcinomas and in healthy adjacent tissues.

Notch-Box plots illustrate the distribution of CD8+ cells (left panel) and PD-1 histoscores (right panel) in gastric and esophageal adenocarcinomas and in corresponding healthy adjacent tissues.


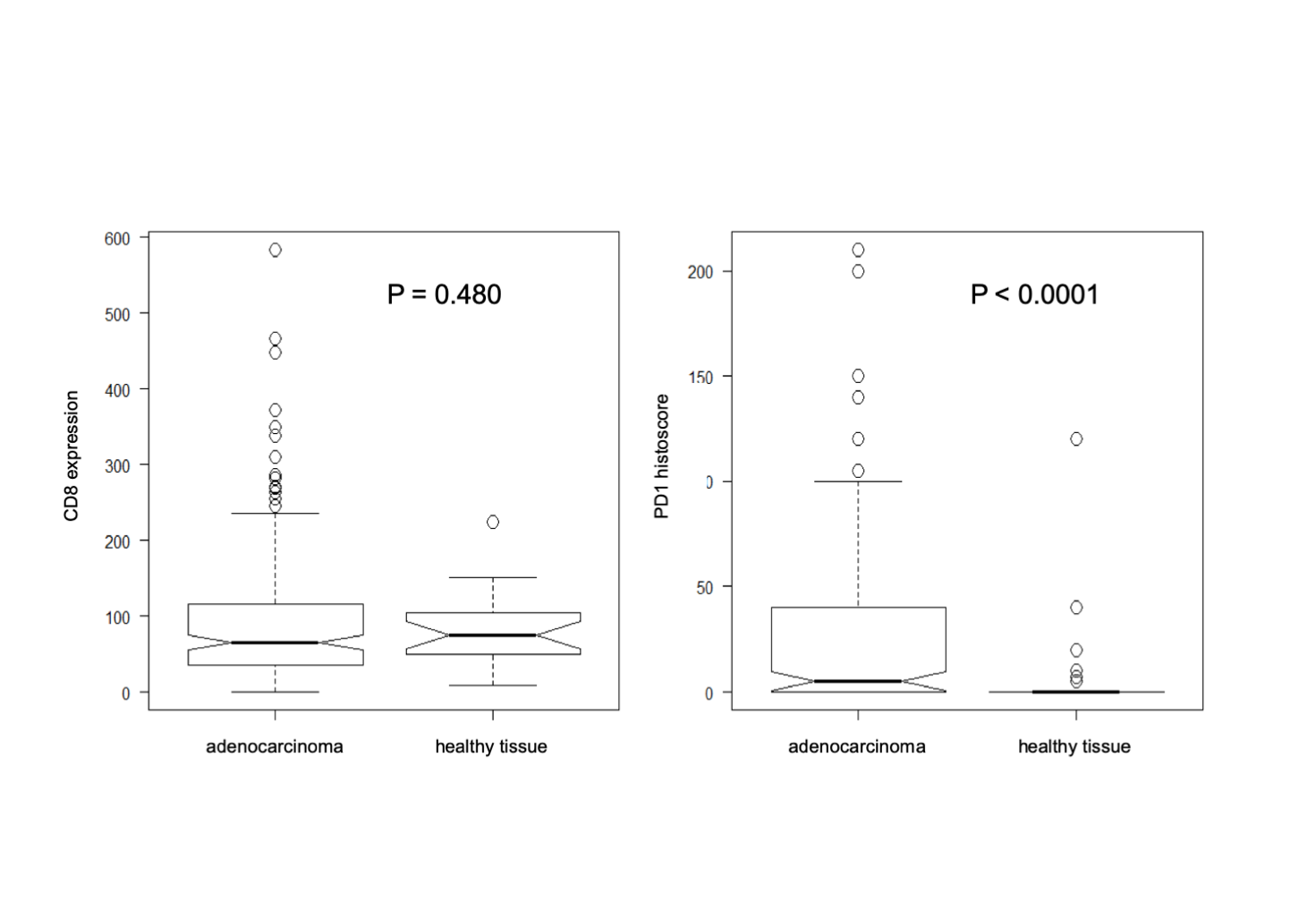


**Supplementary figure 2.** Impact of PD-1/PD-L1/CD8 signature on overall survival in gastric or esophageal adenocarcinoma. Kaplan-Meier curves depict the impact of the consistently high (green line), low (black line), or mixed (red line) expression of the indicated markers in tumor microenvironment on the overall survival of patients with gastric (n=133, P=0.01 panel A) and esophageal (n=28, P=0.03 panel B) adenocarcinomas.

**Supplementary figure 2A**: Prognostic impact of PD1/PD-L1/CD8 signature in gastric adenocarcinoma (n=133; P=0.01).

**
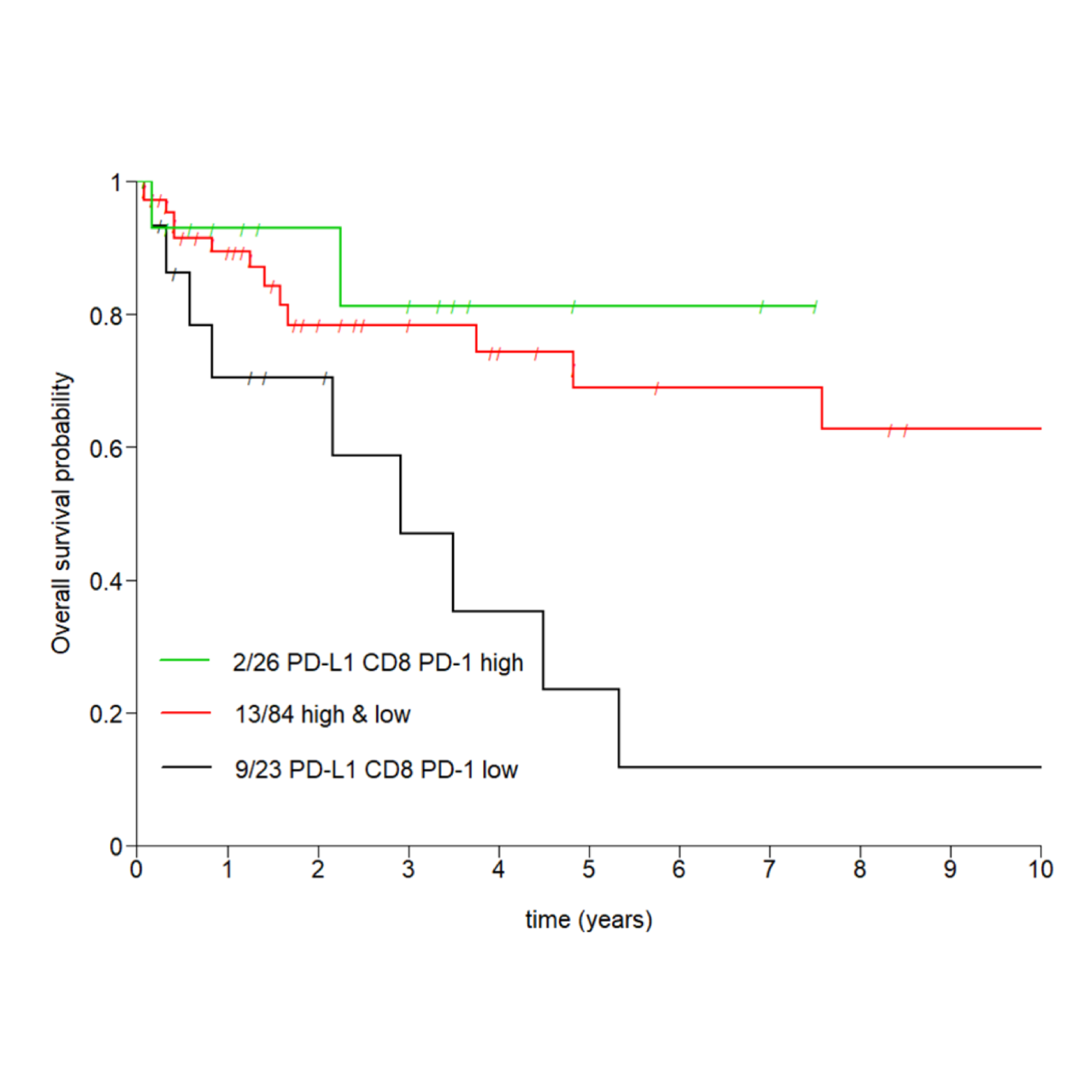
**

**Supplementary figure 2B**: Prognostic impact of PD1/PD-L1/CD8 signature in esophageal adenocarcinoma (n=28; P=0.03)

**
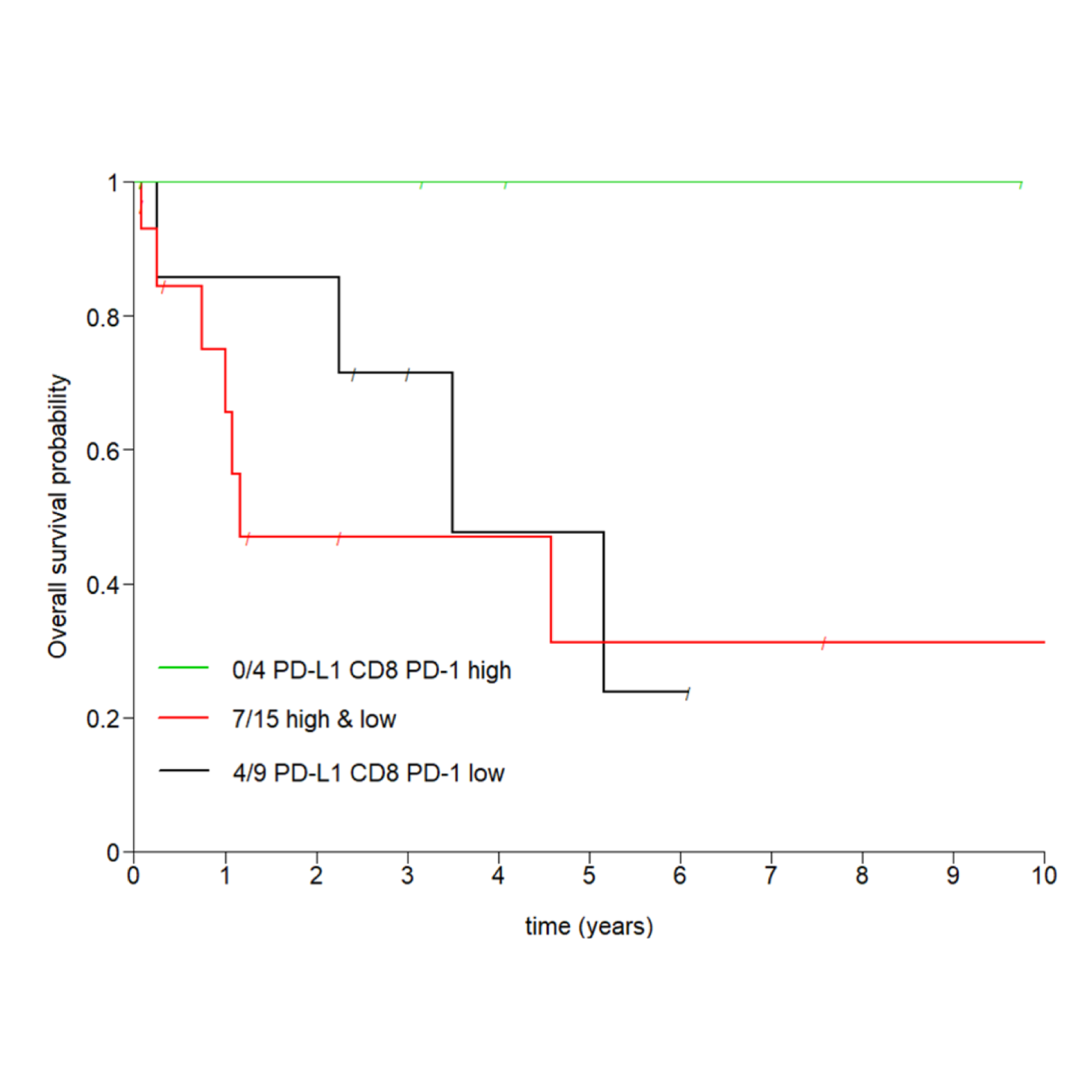
**
